# Interlimb differences in gait kinematics, kinetics, and muscle activation during walking and running one year after acute unilateral Achilles tendon rupture

**DOI:** 10.1186/s13018-025-06221-0

**Published:** 2025-09-15

**Authors:** Kari Huseth, Guðni Rafn Harðarson, Per Aagaard, Annelie Gutke, Roland Zügner, Jón Karlsson, Katarina Nilsson Helander, Elin Larsson, Annelie Brorsson, Roy Tranberg

**Affiliations:** 1 Institute of Clinical Sciences, Department of Orthopedics, Sahlgrenska Academy, University of Gothenburg, Gothenburg, Sweden; 2 Department of Sports Science and Clinical Biomechanics, Muscle physiology and biomechanics Research Unit, University of Southern Denmark, Odense, Denmark; 3 Department of Health and Rehabilitation, Institute of Neuroscience and Physiology, Unit of Physiotherapy, University of Gothenburg, Gothenburg, Sweden; 4 IFK Kliniken Rehab, Gothenburg, Sweden

**Keywords:** Achilles tendon rupture, Neuromuscular activation, Triceps surae, Gait biomechanics, Movement pattern restoration, Motor variability, Muscle-specific training, Rehabilitation

## Abstract

**Background:**

Approximately 30% of individuals with Achilles tendon rupture do not fully restore normal gait function, regardless of the treatment chosen. Limited knowledge exists about the long-term kinematic, kinetic, and neuromuscular adaptations after repair of an acute Achilles tendon rupture and their impact on muscle/tendon function. This exploratory cross-sectional study assessed between-limb differences in terms of lower-limb kinematics, kinetics, and muscle activation during walking and jogging stance phases one year after an Achilles tendon rupture.

**Methods:**

Thirty-seven participants (29 males, 8 females; mean age 47.4 ± 9.4 years) were included in the study one year after Achilles tendon rupture, who were both operatively and non-operatively treated. Electromyography (EMG) was recorded synchronously with kinematic and kinetic data using an optical motion capture system with a cluster-based marker set allowing six degrees of freedom. Bilateral EMG was collected from the tibialis anterior, medial and lateral gastrocnemius, and soleus muscles. The stance phase was divided into initial contact to mid-stance (IC-MS) and mid-stance to toe-off (MS-TO). Differences between affected and unaffected limbs were analyzed with multivariate normal models, reporting point estimates and 95% credible intervals.

**Results:**

During walking, triceps surae activation increased in MS–TO, while running showed greater activation in IC–MS. Affected limbs showed higher lateral gastrocnemius activation during walking IC-MS (2.1 EMG%; CI: 0.5–3.7) as well as greater medial (3.4%; CI: 0.5–6.3) and lateral (4.9%; CI: 2.3–7.6) activation in MS-TO. Ankle sagittal joint excursion was reduced in walking MS-TO (-1.8°; CI: -2.8 to - -0.8) and running MS-TO (-4.1°; CI: -5.8 to -3.5), with decreased sagittal plantarflexor moments during running (0.06 Nm/kg: CI: 0.01–0.11).

**Conclusion:**

One year after Achilles tendon rupture, walking was characterized by increased gastrocnemius muscle activation and reduced ankle sagittal joint excursion compared with the unaffected side. Moreover, running also showed reduced ankle joint excursion accompanied by attenuated plantar flexor moments, however, without any evident side-to-side differences in EMG recordings. Despite the observed inter-limb deficits, gait resembled normative kinematic patterns, likely reflecting compensatory mechanisms. EMG and joint moments were more variable than kinematics. These results support the need for individualized targeted long-term triceps surae rehabilitation following Achilles tendon rupture.

**Supplementary Information:**

The online version contains supplementary material available at 10.1186/s13018-025-06221-0.

## Background

The Achilles tendon is considered the strongest force-transmitting tendon in the human body and plays a critical role in human bipedal locomotion [1]. Its unique architecture includes a 90-degree lateral twist from the myotendinous junction to its calcaneal insertion [1, 2]. The primary function of the triceps surae muscle group is ankle plantarflexion, whereas its antagonist muscle tibialis anterior, serves as the primary dorsiflexor of the ankle. Together these muscle groups enable efficient forward propulsion of the body by integrated actions of the ankle/foot complex [3].

Despite its structural specialization for high-load transmission and energy storage during locomotion, the Achilles tendon remains vulnerable to rupture, with incidence injury rates continuing to rise [4, 5]. In Sweden, the Achilles tendon rupture (ATR) incidence increased from 34.4 to 41.7 and in Finland from 17.3 to 32.3 per 100,000 person-years, with data covering 2017–2021 [4] and 1997–2019 [5], respectively.

Approximately 30% of all ATRs fail to demonstrate restoration towards normal gait function after injury, regardless of surgical or non-surgical treatment [6, 7]. Chronic tendon elongation is observed after ATR in the majority of patients, again irrespectively of surgical or non-surgical treatment, and this elongation may affect the physical performance negatively, especially during activities such as walking and running [8]. After ATR, the gastrocnemius muscle of the affected limb often exhibits reduced muscle volume and shorter fascicle lengths, suggesting both anatomical and functional adjustments [9]. Beyond the direct impact on ankle plantar flexor muscle function, ATR is likely to influence both the kinematics and kinetics of horizontal locomotion, which in turn can lead to compensatory adjustments in movement patterns [10]. As a result, biomechanical (kinematic, kinetic and neuromuscular) changes observed post repair of the torn Achilles tendon are crucial to understand, especially how the body adapts to regain gait function and ensure adequate running performance despite the kinematic, kinetic and neuromuscular changes described above.

Post-operative kinematic data suggest that individuals recovering from ATR display compensatory adaptations during both walking and running. These adaptations are particularly evident during running, where the affected limb shows reduced ankle and knee excursions and lower joint moments, while the unaffected limb often compensates with increased hip motion [11]. Despite these asymmetries, walking kinematics and kinetics appear comparable between individuals who have sustained an ATR and healthy controls, and the unaffected limb demonstrates similar joint mechanics to matched controls during running—indicating that adaptations are primarily confined to the affected side [10].

Thus, delayed heel lift-off has been observed during the walking gait cycle [12] while reduced ankle plantarflexion, decreased joint excursion and increased dorsiflexion during locomotion indicate the adoption of compensatory strategies for propulsion [13]. Running-specific deficits, such as reduced ankle joint excursion from mid-stance to toe-off and knee hyperextension at initial ground contact also have been reported following surgical ATR repair, suggesting altered joint coordination [10]. Additionally, an observed increase in peak dorsiflexion during walking gait stance [14] and decreased knee flexion [15] may reflect efforts to compensate for functional deficits caused by Achilles tendon elongation [16].

Kinetic changes following ATR repair have also been observed, indicating altered load distribution across the ankle, knee and during walking and/or running [10, 11, 17]. Reduced ankle plantar flexion moments and reduced ankle joint power compared with non-injured healthy controls have also been noted during walking [13], accompanied by reduced kinetic outputs during heel-rise testing [17], further suggesting significant chronic impairments in mechanical plantarflexion function following ATR. In addition, observations of increased subtalar eversion moments during jogging and running [15], and reduced ankle joint moments during dynamic tasks such as running, hopping, and jumping [10, 17]. Taken together, these findings suggest an adaptive strategy of compensatory load redistribution to unload the affected ankle joint, potentially increasing mechanical stress on more proximal joints such as the knee and hip—either on the same (ipsilateral) or opposite (contralateral) limb.

Concurrent observations of altered muscle activation patterns suggest the presence of neuromuscular deficits after an ATR [18]. Increased preactivation of the tibialis anterior muscle prior to ground contact along with increased gastrocnemius activation during the push-off phase [18] may indicate compensatory mechanisms to offset reduced plantar flexor strength and smaller active joint excursion in the affected limb. In this context, recent research has highlighted the growing relevance of neuromuscular variability—not only as a marker of adaptation following musculoskeletal injury, but also as an inherent feature of the human motor system thought to support flexible and context-specific movement control [19, 20].

These findings highlight the complex interplay between kinematic, kinetic, and neuromuscular factors in the wake of an ATR recovery. Such alterations are likely to negatively impact body propulsion, thus affecting walking and running efficiency due to impaired muscle force generation and reduced neuromuscular activation in muscles crossing the ankle joint [10, 17, 18]. Despite these insights, only limited knowledge exists about the specific kinematic, kinetic and neuromuscular long-term adaptations in response to ATR and their consequences to regain long-term functional performance.

Thus, the aim of this exploratory cross-sectional study was to investigate the between-limb differences in kinematics, kinetics, and muscle activation patterns of the lower extremities during the stance phase of walking and jogging, one year after an unilateral ATR.

Based on prior research indicating persistent neuromechanical deficits following ATR, it was hypothesized that there would be a probability of systematic between-the-limbs differences in lower extremity biomechanics one year post ATR injury. Specifically, the affected limb was expected to demonstrate reduced joint moments, limited joint excursion, and altered muscle activation patterns compared with the unaffected limb during the stance phase of walking and jogging.

## Material & methods

### Participants

This study was conducted in accordance with the Helsinki declaration and approved by the Swedish Ethical Review Authority (2019–05457). Participants were recruited from the ongoing DUSTAR (Diagnostic Ultrasonography for the Choice of Treatment of Acute Achilles Tendon Rupture) project at the Sahlgrenska Academy, University of Gothenburg, one year (+ 2 months) after the injury. Thirty-seven participants (29 male, and 8 female) with a mean age of 47.4 years (SD ± 9.4) were included in the study (Table 1). The participants reported a mean PAS (Physical activity scale), (range 1–6) of 4.0 (± 1.1), indicating moderate to high physical activity levels [21]. EQ-5D (range 0–1) responses showed moderate to good health at a mean score of 0.7 (± 0.3) [22], .

**Table 1:** Participant demographics and patient reported outcome measurements score (mean (SD))

Age (years)	47.4 (± 9.4)
Height (meter)	1.79 (± 0.08)
Weight (kg)	83.3 (± 16.6)
BMI*	26.9 (± 4.9)
PAS**	4 (± 1.1)
EQ-5D***	0.7 (± 0.3)

* BMI = Body mass index

**PAS = Physical activity scale is a six-graded scale, self-reported level of physical activity, where 1 means extremely low physical activity and 6 means strenuous physical activity

*** EQ-5D is a self-reported measurement score regarding health-related quality of life (range 0–1), 0 = worse than death, 1 = perfect health

Inclusion criteria comprised patients who had sustained an ATR and were treated either surgically or non-surgically. Previous reports have indicated no systematic effects of the treatment protocol (surgical vs. non-surgical) on post-injury motion variables obtained during walking and running [17]. Thus, the two groups were treated as a single cohort, without any comparison. The participants received both written and verbal information prior to signing their consent forms. Data collection took place between September 2021 and March 2023 at the Gait Laboratory at the Orthopedic Research Unit, Sahlgrenska University Hospital, Sahlgrenska Academy, Gothenburg University, Sweden.

Prior to the present measurements, all participants had been engaged in standardized rehabilitation programs offered after acute ATR at various rehabilitation units within the municipal region of Gothenburg and the Västra Götaland Region. Details of the rehabilitation regimen are provided as supplementary information (Additional File 1).

### Instrumentation and procedures

Electromyography (EMG) data (more details provided below) were obtained for the triceps surae muscles (medial gastrocnemius: GM, lateral gastrocnemius; GL, soleus: SOL) and tibialis anterior (TA) in synchronization with concurrent kinematic and kinetic data (Table 2). The movement analysis included 3D kinematic assessments using a motion capture system and multiple force plates embedded into the floor. A cluster-based marker set with six degrees of freedom was applied for the additional movement analysis [17].

**Table 2:** Marker placement for the present 6 degree of freedom model

Anatomical segments	Anatomical markes	Tracking markers
Foot/ankle complex	Distal metatarsal I	Distal metatarsal III
	Distal metatarsal V	Lateral calcaneus
	Medial malleolus	Inferior heel
	Lateral malleolus	Superior heel
Lower leg	Medial tibial condyles	Shank cluster (4x)
	Lateral tibial condyle	
Thigh	Medial femoral condyles	Thigh cluster (4x)
	Lateral femoral condyle	
	Greater trochanter	
Pelvis	Iliac crest	Anterior superior iliac spine
		Posterior superior iliac spine Superior sacrum
Trunk	Acromion	T10 spinal process
		Inferior scapular angle
		Head of sternum
		C7 spinal process

During all lab activities, participants wore standardized footwear (Bagheera Omega, Avesta, Sweden) which was used as the shoe/foot complex for all subsequent analyses.

As a warm-up and practicing period prior to the lab tests, each participant was asked to walk around the lab room for 2–3 min and perform 3–4 submaximal contractions for every given target muscles. Bipolar EMG measurements were conducted according to standard procedures, including skin preparation, electrode placement (Table 3), signal quality evaluation, normalization relative to maximal isometric contraction (MVIC).

**Table 3:** EMG recording in selected lower limb muscles, electrodes positionings

Muscle	Electrode Placements
Medial Gastrocnemius	2 cm apart, parallel to muscle fibers, just distally to the knee, 2 cm medially to the midline of the dorsal aspect of the shank
Lateral Gastrocnemius	2 cm apart, parallel to muscle fibers, just distal to knee and 2 cm lateral to midline of the dorsal aspect of the shank
Medial Soleus	Medially to the Achilles tendon, just below the distal border of medial gastrocnemius.
Tibialis anterior	One-third or one-fourth down the length of the tibia, and Lateral to the tibia, over the greatest muscle mass.

and filtration [23–25]. MVICs were performed for the TA, GM, GL, and SOL in a standardized upright standing position. The feet were secured in a belt apparatus to restrict dorsiflexion and plantarflexion, and participants were strapped to minimize joint motion. Verbal encouragement was provided consistently by the same experienced examiner to ensure maximal effort. A 60-second rest interval was provided between each trial to avoid fatigue. Each muscle’s MVIC value was calculated as the mean of the peak EMG amplitudes from four 6-second contractions. This procedure aligns with established EMG normalization protocols [26, 27].

**Fig. 1:**
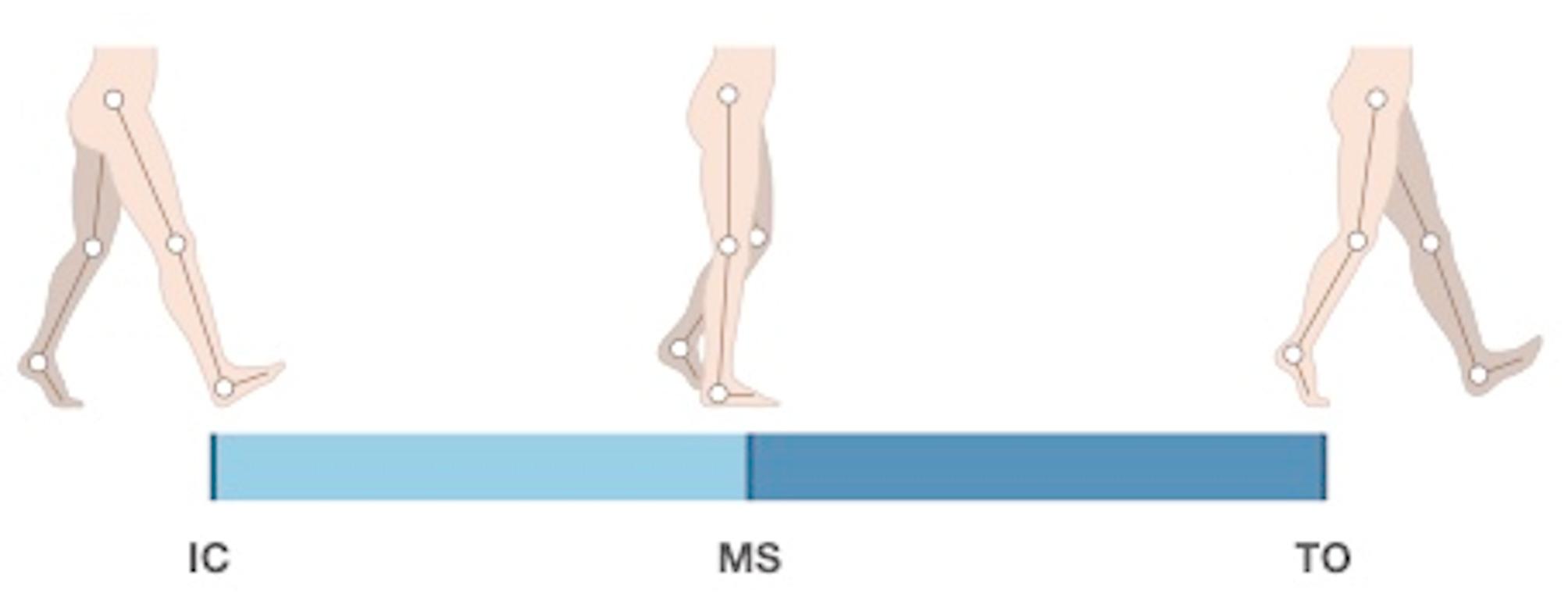
Defined events during the stance phase. IC = initial contact, MS = midstance, TO = toe-off. The white circles represent estimated joint centers at the hip, knee and ankle

Walking and running were performed at self-selected speeds along a 10-meter walkway equipped with four centrally positioned force plates—two on each side—to capture ground reaction forces from both limbs. Gait speed was measured using a set of infrared timing gates (Speedtrap II, Brower Timing Systems, Draper, UT, USA) placed at the start and end of the walkway. The gates were automatically triggered upon entry and exit, and speed was calculated based on the time taken to traverse the 10-meter distance. The resulting mean speeds were 1.36 ± 0.18 m/s for walking and 2.52 ± 0.35 m/s for jogging. Participants completed trials until five successful foot strikes were recorded for each limb during both walking and running, defined as clear, full-foot contacts on the force plates. Each stance phase was divided into three key events—initial contact (IC), midstance (MS), and toe-off (TO)—for subsequent kinetic and EMG analyses (Fig. 1). IC and TO were identified from the force plate data as the time points when the foot first contacted and subsequently left the plate, respectively, while MS was defined as the instant when the center of gravity (COG) passed over the center of pressure (COP).

### Kinetic and kinematic measurements and signal processing

Kinetic and kinematic variables were captured using a marker-based motion capture system (Qualysis AB, Gothenburg, Sweden), featuring 16 infrared cameras (Oqus 7) operating at 250 Hz and synchronized with two video cameras. To measure ground reaction forces, four OPTIMA High-Performance Series force plates (AMTI, Watertown, MA, USA) were utilized, with all force signals recorded synchronously at 1000 Hz A/D sampling rate. During later off-line analysis, the motion capture data (body segment markers) were noise filtered using a 4th-order zero lag low pass Butterworth filter with a cut-off frequency of 10 Hz.

### Electromyography (EMG) recording and signal processing

Pair-wise (bipolar) EMG electrodes were applied to the tibialis anterior, medial and lateral gastrocnemius and medial soleus muscles in a standing position, as outlined in Table 3. EMG signals were recorded bilaterally from both the affected and unaffected limbs.

Electromyographic activity was recorded using the Delsys Trigno Research wireless system with Trigno Avanti electromyography sensors (Delsys Inc, Natic, MA, USA). The system is operated at an A/D sampling frequency of 2000 Hz, to record EMG signals that have initially been filtered through an on-line hardware bandpass filter (25–450 Hz). All EMG signals were amplified using an input impedance of > 1G Ohm, and a common mode rejection ratio of > 80 dB.

Off-line signal processing of the recorded EMG signals involved an initial digital high pass filter with 5 Hz cut-off frequency, to remove potential movement artifacts. The EMG signals were linearly detrended, full-wave rectified, and smoothed using a centered 30-ms root mean square (RMS) window to compute the linear envelope, consistent with standard practice for dynamic tasks [28].

Finally, all EMG signals were normalized relative to the MVIC (i.e. expressed as EMG%). EMG signals recorded in given target muscles were processed using identical EMG filtering procedures for all MVIC trials and walk/run trials.

### Data processing

A six-degree-of-freedom model was set up and data processed using Visual 3D (HAS-Motion, Kingston, Ontario, Canada). The EMG signals were post-processed by calculating the area under the normalized filtered EMG curve, divided by the integration time (to yield the mean average EMG amplitude (%MVIC)). EMG trials were excluded if the signal amplitude exceeded 200% of the participant’s other trials and deviated from the expected muscle activation pattern (e.g., timing, shape, or phase-specific modulation).

Processed data were used to calculate selected kinematic, kinetic, and neuromuscular variables, as detailed in Table 4. The stance phase was divided into two functional sub-phases - initial contact to midstance (IC-MS) and midstance to toe-off (MS-TO) - as illustrated in Fig. 1. EMG activity was expressed as the mean percentage of maximum voluntary isometric contraction (%MVIC) across both sub-phases for the following target muscles: TA, GM, GL, and SOL. Kinematic analysis included joint excursions, calculated in degrees for the ankle, knee, and hip joints in the sagittal plane and for the ankle and hip joints in the frontal plane, separately for IC–MS and MS–TO, capturing the total angular displacement - difference between maximum and minimum joint angles within each sub-phase—for these periods. Kinetic analysis involved calculating mean joint moments (Nm/kg) for the same joints and planes across each sub-phase.

**Table 4:** Variables used in the statistical model analyzing side to side differences in affected and unaffected limbs

Modality	Variable Presented As	Stance Phases	Analyzed Muscles /Planes /Joints and movement direction (+ / –)
EMG	Mean %MVIC	IC–MS MS–TO	MUSCLES: Tibialis anterior (TA) Medial gastrocnemius (GM) Lateral gastrocnemius (GL) Soleus (SOL)
KINEMATICS	Mean joint excursion (°)	IC–MS MS–TO	SAGITTAL PLANE: Hip (extension +/ flexion -) Knee (extension +/flexion -) Ankle (plantarflexion +/ dorsiflexion -) FRONTAL PLANE: Hip (adduction +/abduction -) Ankle (inversion +/eversion -)
KINETICS	Mean joint moment (Nm/kg)	IC–MS MS–TO	SAGITTAL PLANE: Hip (extension +/ flexion -) Knee (extension +/flexion - Ankle (plantarflexion +/ dorsiflexion -) FRONTAL PLANE: Hip (adduction +/abduction -) Ankle (inversion +/eversion -)

Defined periods of gait stance phase: IC-MS = initial contact to midstance, MS-TO = midstance to toe off. EMG: electromyography, MVIC = maximum voluntary isometric contraction

**+** = Extension / Plantar flexion/Adduction/Inversion, **-** = Flexion / Dorsi flexion/Abduction/Eversion

### Statistical analysis

Based on results from a previous study evaluating biomechanical variables during walking, running, and hopping after acute ATR [29], it was estimated that a sample size of 38 patients would be required to detect significant side-to-side differences in lower extremity biomechanics (α = 0.05, power = 80%) at 12 months post-injury.

All processed data were analyzed using a multivariate normal Bayesian model to assess within-subject differences between the affected and unaffected limbs. Point estimates and 95% credible intervals (CI) were defined as the posterior median and the 2.5th and 97.5th percentiles of the posterior sample, respectively. In this model, posterior median and mean are not distinguishable; thus, descriptive statistics are reported as means and were generated using IBM SPSS Statistics (v. 30.0).

Initially, separate multivariate models were fitted for kinetic, kinematic, and EMG data respecively, analyzed independently for walking and running. Complete case data were used for all models. As a sensitivity analysis, each outcome variable was also modeled separately using univariate linear models that included only an intercept and standard deviation.

Models were implemented in R (version 4.5.0) [30], using the brms package (version 2.21.0) [31], which provides an interface to Stan for Bayesian inference. Bayesian estimation was based on the joint posterior distribution, which allowed for quantification of uncertainty in all parameters, including average side-to-side differences, population standard deviations, and correlations between variables.

To fit the models, six Markov chain Monte Carlo (MCMC) chains were run in parallel, yielding 6,000 post–warm-up iterations. Convergence was evaluated using the improved R-hat statistic [27]. Default weakly informative priors were applied: a Student’s t-distribution with 3 degrees of freedom for intercepts, a half-Student’s t-distribution for standard deviations, and an LKJ prior for covariance matrices. These priors introduce mild regularization, slightly shrinking the posterior toward zero and thereby reducing the risk of type I and type M errors when dichotomizing credible intervals.

Quantitatively similar results can be obtained using penalized frequentist regression techniques such as ridge regression or LASSO. Model comparisons were conducted using the expected log pointwise predictive density (ELPD) [32], . Side-to-side differences are presented as posterior point estimates with corresponding 95% CI.

In addition, the coefficient of variation (CV) was calculated as the ratio of the standard deviation to the mean to descriptively assess within-subject variability in kinematic, kinetic, and EMG parameters.

## Results

**Fig. 2:**
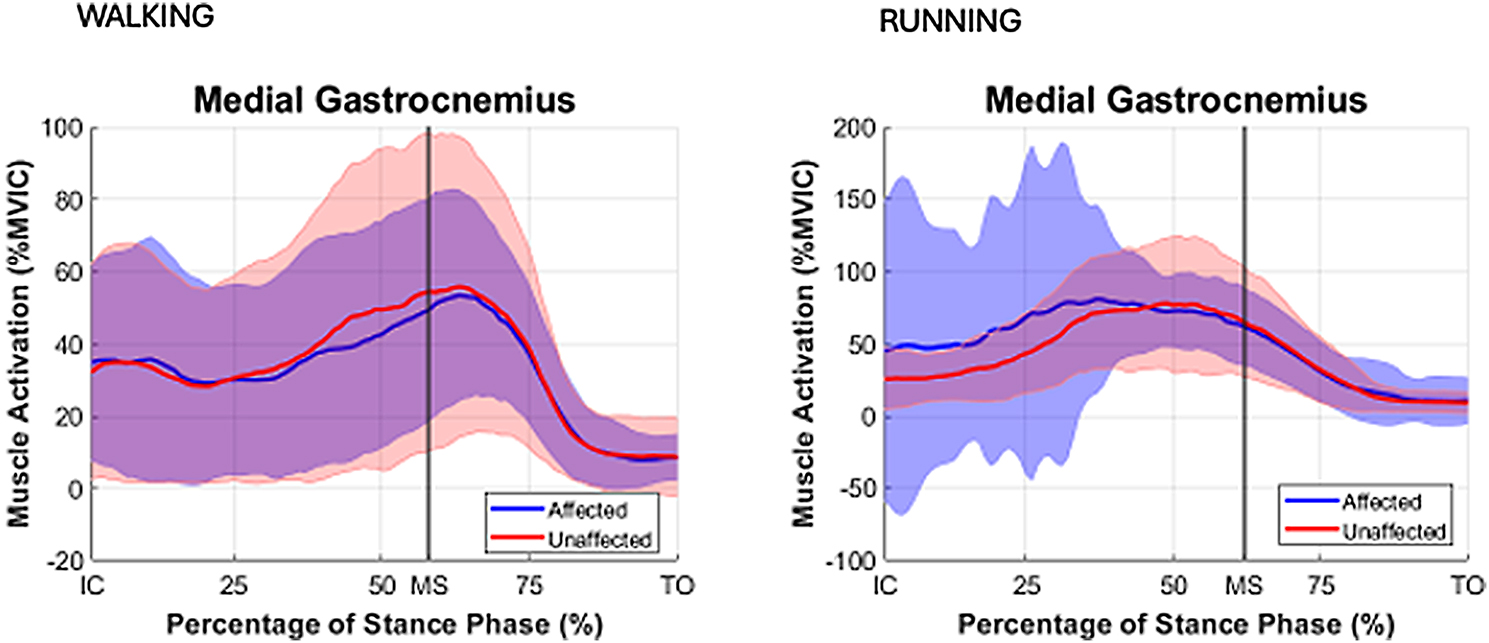
Mean EMG % MVIC distribution curves with SD for stance phase for muscle medial gastrocnemius (GM) for the affected (blue) and for the unaffected (red). limbs Stance phase events: IC = initial contact, MS = midstance, TO = toe off

**Fig. 3:**
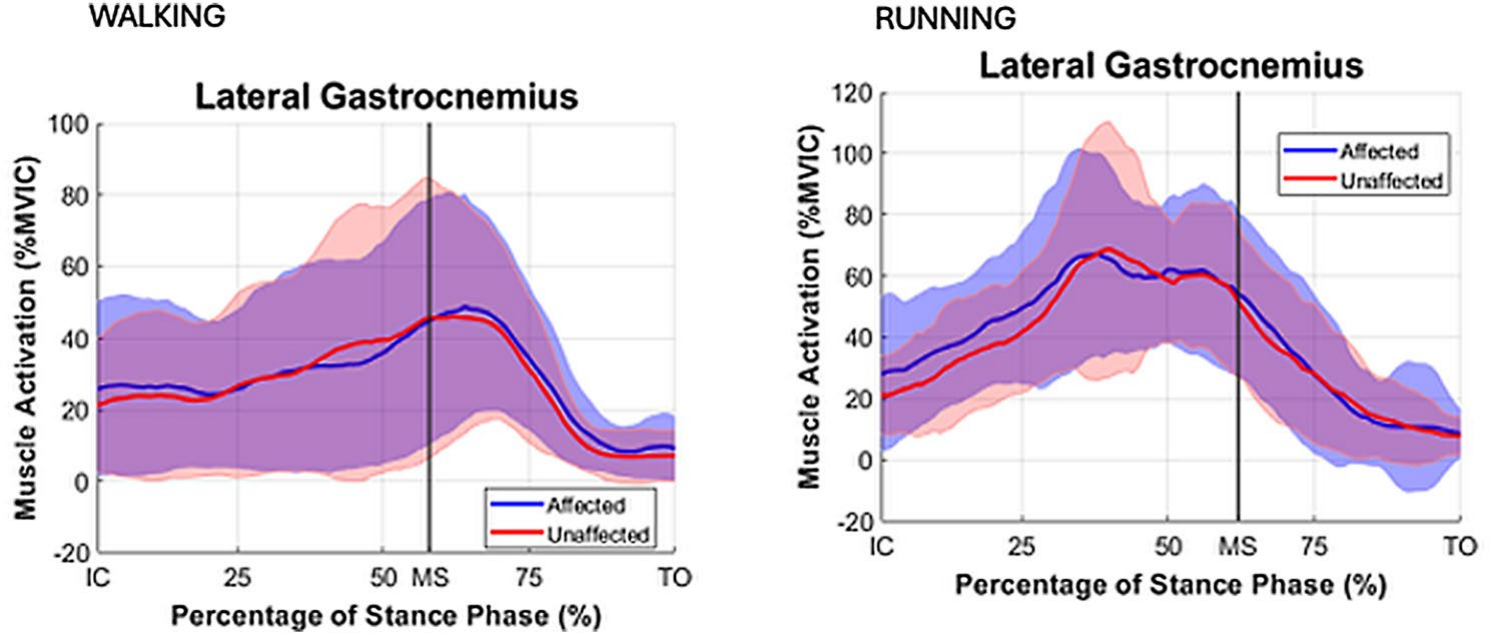
Mean EMG % MVIC distribution curves with SD for stance phase for muscle lateral gastrocnemius (LG) for the affected (blue ) and unaffected (red) limbs. Stance phase events: IC = initial contact, MS = midstance, TO = toe off

**Fig. 4:**
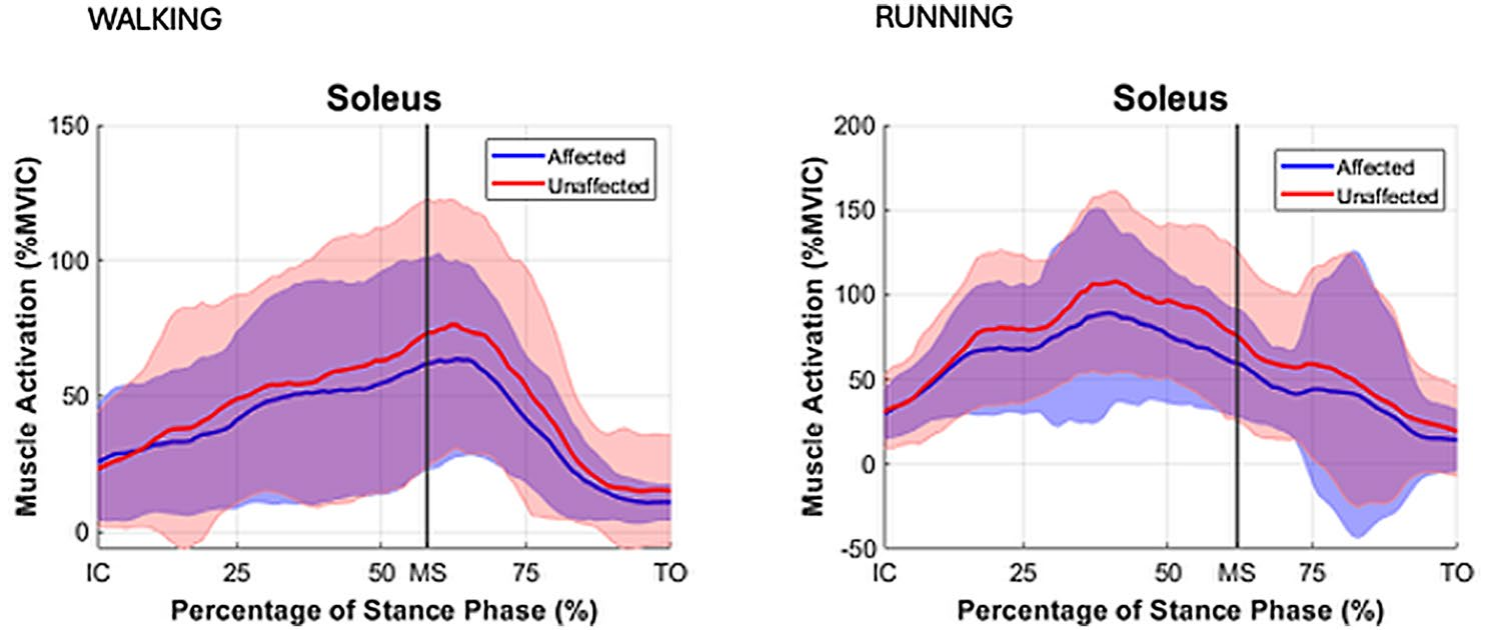
Mean EMG % MVIC distribution curves with SD for stance phase for muscle soleus (SOL) for the affected (blue) and unaffected (red) limbs. Stance phase events: IC = initial contact, MS = midstance, TO = toe off

During both walking and running, all examined muscles were observed to exhibit higher EMG activity during running, with the most pronounced differences observed in the triceps surae group. For the TA muscle activity during IC-MS phase increased from walking to running on both the affected (11% vs. 20% EMG% and unaffected sides (12% vs. 18%). During the MS-TO, the TA activity remained low across both gait modes, increasing slightly from walking to running on the affected (6% vs. 12%) and unaffected sides (7% vs. 11%). For the GM muscle during IC-MS, EMG% increased from 13 to 54% on the affected side and from 11 to 54% on the unaffected side. A similar pattern was observed during MS-TO, with EMG activity increasing from 21 to 29% on the affected side and from 19 to 34% on the unaffected side. GL followed a comparable trend. During IC-MS, EMG activity increased from 9 to 53% on the affected side and from 7 to 52% on the unaffected side. In the MS-TO phase, EMG activity increased from 19 to 29% on the affected side and from 16 to 29% on the unaffected side. SOL showed the most pronounced increase in activation between waking and running. During IC-MS, EMG activity increased from 7 to 78% on the affected side and from 19 to 81% on the unaffected side. During MS-TO, soleus EMG activity increased from 33 to 40% on the affected side and from 33 to 49% on the unaffected side. (Table 5; Figs. 2, 3 and 4)

**Table 5:** Descriptive EMG data from affected and unaffected limbs during gait and running (*N* = 37)^a^

EMG							MUSCLES	
LOCOMATION MODE	TIBIALIS ANTERIOR	TIBIALIS ANTERIOR	MEDIAL GASTROC	MEDIAL GASTROC	LATERAL GASTROC	LATERAL GASTROC	SOLEUS	SOLEUS
GAIT	AFFECTED	UNAFFECTED	AFFECTED	UNAFFECTED	AFFECTED	UNAFFECTED	AFFECTED	UNAFFECTED
IC-MS	10.45 (± 6.78)	11.47 (±6.08)	12.85 (±7.34)	10.95 (±8.00)	8.58 (±5.86)	6.78 (±3.53)	6.78 (±3.53)	19.28 (±9.62)
MS-TO	6,16 (±4.99)	6,55 (±6.00)	21.37 (±9.54)	19.19 (±11.02)	19.40 (±10.83)	15.62 (±6.65)	33.00 (±14.93)	32.88 (±17.60)
RUN	AFFECTED	UNAFFECTED	AFFECTED	UNAFFECTED	AFFECTED	UNAFFECTED	AFFECTED	UNAFFECTED
IC-MS	19.62 (±26.14)	17.75 (±13.34)	53.85 (±26.11)	53.82 (±31.31)	53.35 (±24.57)	51,97 (±22.80)	78,38 (±42.94)	80.90 (±44.50)
MS-TO	11.52 (±10.73)	11.36 (±8.87)	28.97 (±15.67)	33.83 (±19.39)	29.10 (±18.80)	29,45 (±14.14)	39,91 (±22.95)	49.27 (±41.65)

^a^Data are presented as mean (± SD) % MVIC

**Fig. 5:**
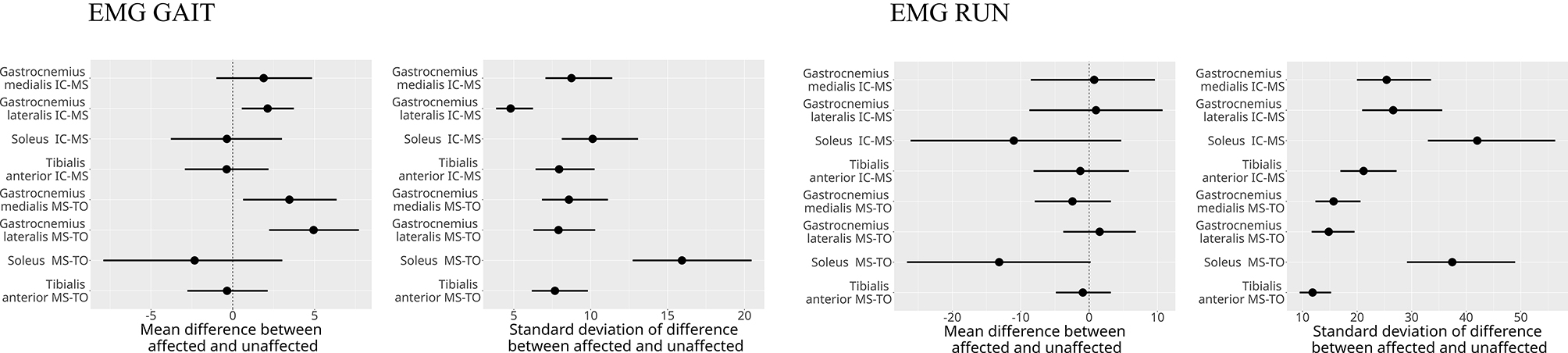
Posterior point estimates of interlimb differences in EMG amplitude (%MVIC) during stance phases of walking and running. IC—MS = initial contact mid-stance, MS-TO = mid-stance to toe-off. Error bars represent 95% credible intervals. *Posterior estimates indicated credible interlimb differences in muscle activation during walking. For the gastrocnemius lateralis, the estimated difference during IC—MS was 2.1 EMG % (CI: 0.5, 3.7). During MS-TO, the gastrocnemius medialis showed a difference of 3.4 EMG % (CI: 0.6, 6.3) and the gastrocnernius lateralis 4.9 EMG % (CI: 2.3, 7.6). These intervals exclude zero, indicating strong posterior evidence Of increased activation in the unaffected limb

**Fig. 6:**
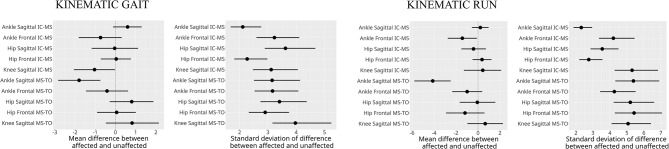
Posterior point estimates of interlimb differences in joint excursion during stance phases Of walking and running. IC-MS = initial contact to mid-stance, MS-TO = mid-stance to toe-off. Error bars represent 95% credible intervals. *Posterior estimates indicated credible interlimb differences in ankle joint excursion in the sagittal plane. During walking, the estimated difference during MS—TO was − 1.8° (CI: -2.8, -0.7); during running, the difference during MS-TO was − 4.1° (CI: -5.8, -3.5). These intervals exclude zero, indicating reduced ankle joint excursion in the affected limb

During walking, posterior estimates indicated greater GL activation on the affected side compared to the unaffected side during IC- MS, with a mean difference of 2.1 EMG% and a 95% credible interval (CI) of 0.5 to 3.7 (6). Similarly, during the second half of the stance phase (MS- TO), the affected limb showed higher activation in both GM (mean difference: 3.4 EMG% 95% CI: 0.6 to 6.3) and GL (mean difference: 4.8 EMG%, 95% CI: 2.3 to 7.6), relative to the unaffected side (Fig. 5). In addition, ankle sagittal joint excursion was likely reduced on the affected side during walking, with a posterior mean difference of -1.8°, and a 95% credible interval ranging from − 2.8° to − 0.7° (Fig. 6).

**Fig. 7:**
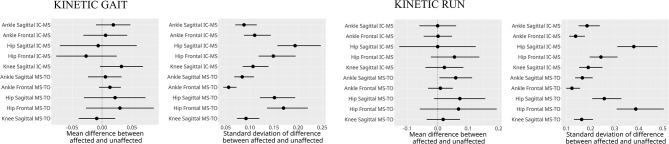
Posterior point estimates of interlimb differences in joint moments (Nm/kg) during stance phases of running. IC-MS = initial contact to mid-stance, MS-TO = mid-stance to toe-off. Error bars represent 95% credible intervals. *Posterior estimates indicated a credible interlimb difference in ankle joint moment in the sagittal plane during MS—TO: 0.06 Nm/kg moment (CI: 0.01–0.11). The credible interval excludes zero, providing strong evidence of reduced knee joint moment in the affected limb during MS-TO in running

During running, the affected limb also showed reduced ankle sagittal joint excursion during the MS-TO phase (mean difference: − 4.1°, 95% CI: − 5.8° to − 2.5°), accompanied by a lower ankle sagittal moment (mean difference: − 0.06 Nm/kg, 95% CI: − 0.11 to -0.01) compared with the unaffected side (Figs. 6 and 7).

For variables in the frontal plane, no differences between the affected and unaffected limbs were observed, neither during walking nor running (Figs. 6 and 7).

## Discussion

One year post ATR, various biomechanical changes in the shank complex were observed in terms of lower limb muscle activation and kinematics in the sagittal plane. As demonstrated by the present kinematic data, the inter-individual variance in ankle, knee and hip joint excursion was low, whereas for the joint moment and EMG data, higher levels of inter-individual variance were observed.

The observation of increased neuromuscular activation of the MG and LG muscles of the affected versus non-affected side during horizontal walking aligns with previous findings in persons affected by unilateral ATR, which have demonstrated elevated GL activation in the affected limb during heel rise tests, unilateral hopping and walking [18, 33–35], in combination with signs of plantar flexor weakness [36]. The present observation of reduced ankle joint excursion in the sagittal plane during stance is also consistent with earlier reports [37] altogether demonstrating impaired sagittal-plane ankle mobility and related negative impacts on gait performance. Notably, no differences in joint moment during the stance phase of walking were observed between the affected and unaffected limbs (Fig. 7).

**Fig. 8:**
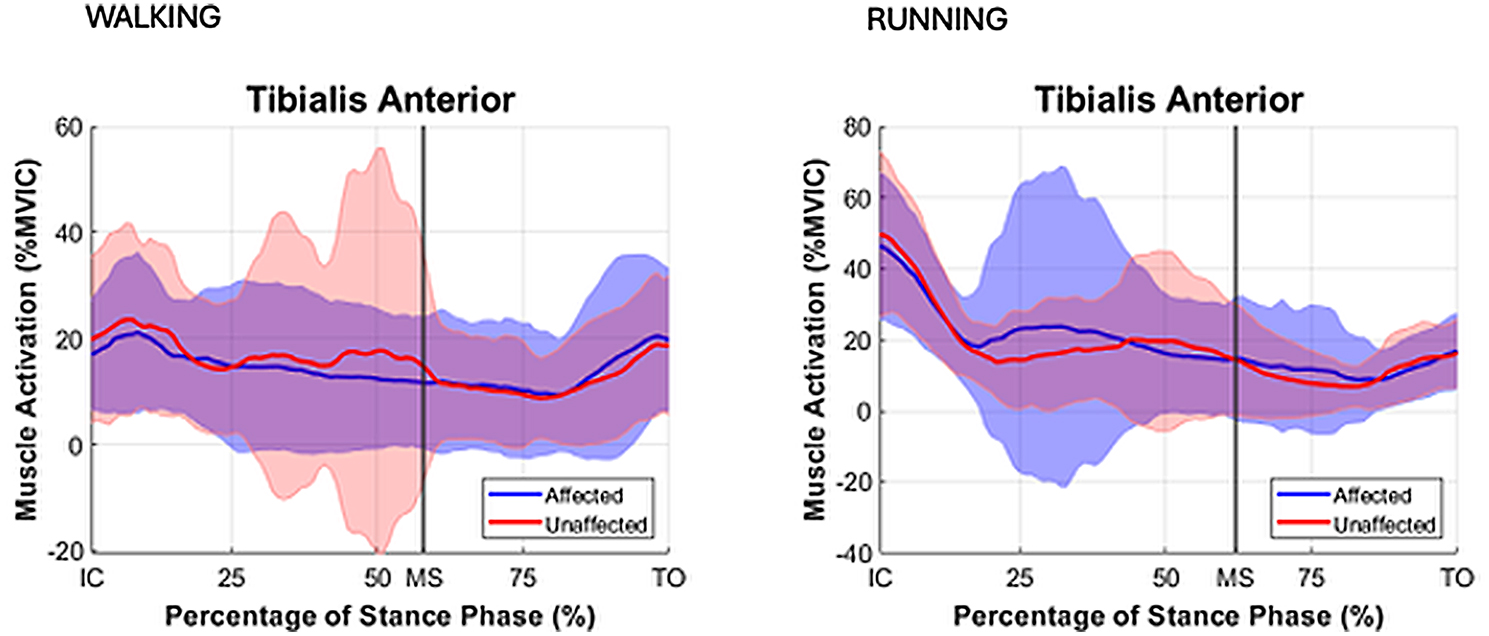
Mean EMG % MVIC distribution curves with SD for stance phase for muscle tibialis anterior (TA) for affected (blue) and unaffected (red) limbs. Stance phase events: IC = initial contact), MS = midstance, TO = toe off

During running, GM, GL and SOL demonstrated peak muscle activation during the phase of IC-MS, as opposed to walking where peak activation emerged during the later phase of midstance to toe-off (MS-TO). Peak activation in the TA muscle was found to occur during the initial stance phase (IC–MS) in both running and walking, irrespective of whether the limb was affected or unaffected (Figs. 8, 2, 3 and 4). This shift in peak muscle activation likely reflects the differing biomechanical strategies employed in each locomotion mode. Walking primarily utilizes an inverted pendulum mechanism during stance, whereas running relies on a more elastic, bouncing strategy [38, 39]. Such divergent mechanical demands can influence the functional contribution of the triceps surae before and after midstance for the two different locomotion modes.

As a main finding in the present study, plantar flexor muscle activation was found to be elevated during walking in the affected limb for GL (entire stance phase) and GM (second half of the stance phase) compared to the contralateral non-affected limb (Fig. 5). In contrast, no between-limb differences were observed in soleus activation. Notably, identical findings recently were reported one year after ATR, demonstrating higher GL EMG amplitudes on the injured vs. non-injured side during walking, whereas no side-to-side differences could be observed for the soleus [35]. Taken together, these observations suggest a consistent shift from monoarticular (SOL) to biarticular (GL, GM) plantarflexor activation during walking in long-term response to ATR, potentially reflecting an increased proximo-distal transport of energy (power) from the knee extensors to the plantarflexors as a compensatory mechanism [40–42].

Unlike walking, running did not show apparent differences in lower leg muscle activation between affected and unaffected limbs. Nonetheless, both sagittal ankle joint excursion and sagittal ankle joint moments were reduced in the affected limb compared with the unaffected limb after midstance during running (Figs. 6 and 7). The decreased plantarflexor moment may be attributed to reduced muscle mass (and resulting reduced force output) in the triceps surae muscle of the affected limb [35, 43] and/or potentially caused by structural changes in the Achilles tendon following rupture and repair, the latter including tendon elongation [44–46], reduced stiffness [47], impaired force transmission, or disrupted muscle–tendon interaction. Such muscular and tendonous impairments would be likely to compromise the mechanical efficiency of the triceps surae complex during the propulsive phase of the gait cycle. The lack of side-to-side differences in EMG during running, while apparent during walking gait could also be due to the benefit of the higher momentum in the running vs. walking condition, which aids the forward propulsion and thus decrease the need for muscular effort [38, 48].

Running speed in the present cohort was relatively slow; thus it would be of interest to investigate whether triceps surae activation would be more influenced at higher running velocities, where greater force generation and amounts of energy storage and elastic recoil in the Achilles tendon would be expected.

The observation of small standard deviation (SD) values in the present kinematic data suggests relatively modest inter-individual variability in ankle, knee, and hip joint excursion patterns during the stance phase of both walking and running, as reflected by CV ranging from 9 to 38% for walking and 16–57% for running. Whereas, the EMG results and kinetic data demonstrated markedly higher inter-individual variability, with CVs ranging from 38 to 92% for EMG during walking and from 46 to 136% during running. Joint moment data showed the greatest variability, with CVs ranging from 9 to 900% during walking and from 17 to 660% during running (Table 7).

These findings suggest substantial inter-individual variability in neuromuscular activation and joint kinetics, in contrast to the more consistent joint kinematics observed across participants. This indicates that, although individuals with unilateral ATR may exhibit relatively uniform joint movement patterns during the stance phase of walking and running, such consistency is achieved through considerable variability in shank muscle activation patterns and in the distribution of joint moment generation across the lower limb.

Although no significant differences in soleus activation patterns were observed between the affected and unaffected limbs in the present study, to the data show a greater inter-individual variability manifested by greater SD (Table 5) and larger CI in the EMG distribution patterns (Figs. 4 and 5) obtained for the affected limb compared to the unaffected limb, particularly in the late stance phase (MS -TO) during running where CV values for muscle activation in the affected limb were 46–136% in comparison to CV values in the unaffected limb of 43–85%. Regardless, the large range of variance suggests substantial heterogeneity in muscle activation strategies among individual persons who are affected by ATR in the long term, reflecting the presence of individualized neuromuscular compensatory adaptations following an ATR.

The soleus muscle, composed predominantly of type I fibers (~ 70–85%), primarily supports posture during gait in healthy adults [49]. After ATR, structural changes have been observed in the soleus muscles including increased adipose infiltration and altered fascicle architecture, altogether suggesting functional remodeling of this monoarticular plantar flexor muscle in long-term response to ATR [50–52]. These changes may shift the soleus from a primarily postural role, to gain a more dynamic function [53], while muscles like the lateral gastrocnemius (~ 50% type II fibers) [49], conversely may adapt to serve more stabilizing function. Similar muscle function transitions have been observed in paraspinal muscles post-injury [54, 55]. Along with Achilles tendon elongation and altered twist [56], such adaptations may profoundly alter function of the soleus muscle during horizontal locomotion activities.

The present cohort demonstrated a considerable degree of functional recovery following Achilles tendon injury, generally demonstrating a high degree of symmetry observed between the kinematic output of the affected and unaffected limbs (Table 6). Thus, compensatory mechanisms across the musculoskeletal and motor control systems appear to have been engaged to restore movement patterns that approximate the healthy state of pre-injury symmetry [57].

**Table 6:** Descriptive kinematic data from affected and unaffected limb during gait and running (*N* = 37)^a^

KINEMATICS		JOINT RANGE OF MOTION								
LOCOMATION MODE	ANKLE SAGITTAL	ANKLE SAGITTAL	ANKLE FRONTAL	ANKLE FRONTAL	KNEE SAGITTAL	KNEE SAGITTAL	HIP SAGITTAL	HIP SAGITTAL	HIP FRONTAL	HIP FRONTAL
GAIT	AFFECTED	UNAFFECTED	AFFECTED	UNAFFECTED	AFFECTED	UNAFFECTED	AFFECTED	UNAFFECTED	AFFECTED	UNAFFECTED
IC-MS	13.3 (±2.0)	12.7 (± 2.0)	6.6 (± 2.5)	7.4 (± 2.4)	15.1 (± 3.9)	16.1 (± 3.0)	28.6 (± 3.2)	28.6 (± 4.1)	7.7 (± 2.9)	7.6 (± 2.4)
MS-TO	24.7 (± 3.6)	26.5 (± 3.4)	7.3 (± 2.1)	7.8 (± 2.7)	43.6 (± 3.9)	42.8 (± 3.4)	15.5 (± 3.2)	14.7 (± 3.3)	12.3 (± 2.6)	12.2 (± 2.8)
RUN	AFFECTED	UNAFFECTED	AFFECTED	UNAFFECTED	AFFECTED	UNAFFECTED	AFFECTED	UNAFFECTED	AFFECTED	UNAFFECTED
IC-MS	16.8 (± 2.8)	16.6 (± 2.7)	10.6 (± 3.7)	12.0 (± 3.9)	27.5 (± 5.5)	27.1 (± 4.3)	10.1 (± 4.8)	10.5 (± 4.5)	5.0 (± 2.4)	4.6 (± 2.6)
MS-TO	38.3 (± 6.3)	42.3 (± 5.0)	8.03 (± 3.1)	8.9 (± 3.5)	22.4 (± 5.0)	21.7 (± 5.8)	28.0 (±6.6)	28.2 (± 6.1)	8.7 (± 4.2)	9.8 (± 3.7)

^a^Data are presented as mean (± SD) in degrees

**Table 7:** Descriptive kinetic data from affected and unaffected limbs during gait and running (*N* = 37)^a^

KINETICS			JOINT MOMENT							
LOCOMATION MODE	ANKLE SAGITTAL	ANKLE SAGITTAL	ANKLE FRONTAL	ANKLE FRONTAL	KNEE SAGITTAL	KNEE SAGITTAL	HIP SAGITTAL	HIP SAGITTAL	HIP FRONTAL	HIP FRONTAL
GAIT	AFFECTED	UNAFFECTED	AFFECTED	UNAFFECTED	AFFECTED	UNAFFECTED	AFFECTED	UNAFFECTED	AFFECTED	UNAFFECTED
IC-MS	-0.09 (±0.12)	-0.11 (±0.10)	0.02 (±0.08)	0.01 (±0.09)	-0.36 (±0.13)	-0.38 (±0.12)	-0.51 (±0.17)	-0.50 (±0.18)	-0.51 (±0.22)	-0.48 (±0.15)
MS-TO	-0.97 (±0.11)	-0.98 (±0.09)	0.05 (±0.04)	0.04 (±0.05)	-0.05 (± 0.11)	-0.03 (± 0.11)	-0.33 (±0.16)	0.31 (±0.18)	0.33 (±0.14)	0.31 (±0.12)
RUN	AFFECTED	UNAFFECTED	AFFECTED	UNAFFECTED	AFFECTED	UNAFFECTED	AFFECTED	UNAFFECTED	AFFECTED	UNAFFECTED
IC-MS	-0.79 (± 0.25)	-0.79 (±0.26)	0.08 (±0.11)	0.08 (±0.12)	-1.25 (±0.23)	-1.28 (±0.22)	-0.93 (±0.33)	-0.93 (±0.45)	-0.65 (±0.19)	− 0.070 (±0.25)
MS-TO	-1.30 (±0.25)	-1.36 (±0.24)	0.17 (±0.09)	0.16 (±o.09)	-0.50 (±0.21)	-0.51 (±0.19)	0.12 (±0.21)	0.05 (±0.26)	0.12 (±0.30)	0.05 (±0.33)

^a^Data are presented as mean (± SD) in Nm/kg

The high degree of inter-limb symmetry in kinematic output presenty observed may, at least in part, be attributable to the structured and coherent rehabilitation strategy implemented for this cohort. However, the observed inter-individual variability—particularly evident in EMG signals and kinetic profiles—suggests that individual participants likely adopted a unique neuromuscular strategy for muscle activation and force generation during walking and running. This variability emphasizes the personalized nature of motor control and recovery following ATR [20, 58].

The present study had several limitations that should be considered when interpretending the reported data. Firstly, the relatively small sample size may have limited a sufficient statistical power to detect subtle effects between affected and unaffected limbs, increasing the risk of statistical type-II error. Secondly, the comparison between the affected and unaffected limbs introduces potential confounding factors as locomotion is inherently a bilateral and cyclic activity. Given the body’s natural tendency to restore symmetry through compensatory mechanisms [59], the unaffected limb may itself exhibit adaptive changes, thereby complicating the interpretation of limb-to-limb comparisons. Moreover, the absence of a healthy normative control group limits the ability to determine whether observed patterns reflect pathological alterations or functional adaptations. Investigating GRF-based load redistribution in relation to compensatory mechanisms, both within and between limbs, represents a promising avenue for future research. Finally, the present cohort was recruited from a larger ongoing study, which may introduce sampling bias, as participants may not be fully representative of the broader post-injury population.

The distribution curves of biomechanical variables during the stance phase for both the affected and unaffected limbs (Figs. 8, 2, 3 and 4) were consistent with normative gait data previously reported by Winter et al. [60] and Simonsen et al. [61], based on small cohort studies. To our best knowledge, no recent publications exists providing directly comparable data. This gap may be attributed to the challenges associated with normalizing gait data across individuals, the substantial workload required for comprehensive gait laboratory assessments of multiple variables, and the necessary sample sizes to ensure sufficient statistical power, while correcting for multiple comparisons. Consequently, it appears essential in future research studies to develop up-to-date databases that enable meaningful comparisons of locomotor recovery in individuals with lower limb injuries.

From a rehabilitation perspective, future research efforts should aim to develop targeted training protocols that address the individual components of the triceps surae complex, with the goal of restoring muscle-specific function and optimizing overall lower limb performance during self-supported overground locomotion.

## Conclusion

One year after acute ATR, patients showed increased GL activation before and after mid-stance, and elevated GM activation after-mid-stance (MS-TO) in the affected limb. During the latter stance phase (MT-TO), sagittal plane ankle joint excursion was reduced on the affected side during both walking and running, alongside a further decrease in ankle joint moment during running. Despite these deficits, overall stance-phase biomechanics resembled normative gait, suggesting that effective compensatory adaptations have occurred. The present findings also demonstrate inter-individual variability in terms of neuromuscular activation and joint kinetics, contrasting with the more consistent joint kinematics observed across participants. The lack of updated normative gait data limits interpretation of the present observations, underscoring the need for expanded and revised benchmarks. From a rehabilitation perspective, the results support implementing individualized muscle-specific protocols post-ATR—particularly targeting the triceps surae—to restore function and optimize gait and running performance.

## Supplementary Information

Below is the link to the electronic supplementary material.

Supplementary Material 1

## Data Availability

Data is provided within the manuscript or supplementary information files.
