# Supplementary material for: Interlimb differences in gait kinematics, kinetics, and muscle activation during walking and running one year after acute unilateral Achilles tendon rupture
Source: J Orthop Surg Res. 2025 Sep 15;20:819. doi: 10.1186/s13018-025-06221-0 (PMC12439372; doi:10.1186/s13018-025-06221-0)
Supplement: Supplementary file 1 — Supplementary Material 1 [file 13018_2025_6221_MOESM1_ESM.docx]

**Sahlgrenska University Hospital – Occupational Therapy and Physiotherapy**

**Implementation / Physiotherapeutic Treatment Plan Following Acute Achilles Tendon Rupture**
**Applicable to both surgical and non-surgical management**

**Emergency Department (Initial Management):**

- The affected foot is immobilized in a cast in plantarflexion.
- Provide the patient with the brochure “Information for Patients with Achilles Tendon Injury” and review the prescribed exercises.
- Advise the patient to avoid placing the foot on the floor whenever possible; the foot should be kept elevated in a horizontal position during rest to minimize swelling.
- When standing (e.g., for hygiene, dressing, or food preparation), the foot may rest lightly on the floor for balance.
- The patient should off-load the injured leg using two crutches.
- Gait should involve active hip and knee movement on the affected side without weight-bearing on the foot.
- Patients with impaired balance may require a walking aid (e.g., Beta crutch), and in some cases, a wheelchair. Toe-touching the ground with the injured foot may aid in balance.

**2 Weeks Post-Injury:**

- At the orthopedic outpatient follow-up, the cast is replaced with an orthosis, which must be worn at all times, including during showering.
- Instruct in the home exercise program "Achilles Tendon Program I."
- Continue to encourage toe mobility.
- Educate the patient to routinely palpate the tendon to monitor for signs of overload (increased warmth, redness, swelling).
- Teach how to remove and reapply the orthosis for hygiene and exercise purposes.
- Encourage regular ventilation of the lower leg and provide instructions on cleaning the foot and leg with a washcloth.
- Fit the orthosis with three wedges as appropriate (typically smaller wedges). Patients may add a personal insole or silicone heel cup for comfort.
- If tension is felt in the tendon during gait with three wedges, a fourth wedge may be added temporarily and removed by the patient after several days based on comfort.
- Begin full weight-bearing gait training with two crutches.
- Practice gait on flat surfaces and stairs if applicable, ensuring no uncomfortable stretching of the tendon.
- Recommend a cork wedge in the contralateral shoe to equalize leg length. For patients with special needs, refer to an orthopedic technician for shoe modification.
- Begin planning for appropriate footwear for use post-orthosis. Indoor and outdoor shoes should have a heel height differential of at least 1.5 cm. Flat shoes (e.g., sneakers) are not appropriate; both pairs should have a heel counter. Shoes can be assessed at the next visit.
- Plan for continued physiotherapy. If referred to primary care, ensure the receiving provider is aware of their role in early rehabilitation and responsible for progressive wedge and orthosis weaning, starting at 8 weeks post-injury.

**4 Weeks Post-Injury:**

- Follow-up with treating physiotherapist.
- Review "Achilles Tendon Program I."
- Assess the tendon: continuity, width, consistency.
- Perform the Thompson test.
- Evaluate current tendon loading; if needed, reduce walking volume.
- Remove the lowest wedge.
- Retrain gait on flat and stair surfaces.
- Wean off crutches indoors if gait is well-balanced and the tendon is not under excessive strain.
- Assess footwear. Heel elevation should remain ≥1.5 cm. Shoes must have a heel counter.
- Determine frequency and location of continued rehab.
- **Surgical patients**: orthosis can be removed at night and for seated showering (non-weight-bearing).
- **Non-surgical patients**: orthosis must remain on 24/7.
- For surgical cases: monitor wound healing and begin scar mobilization once healed.
- All patients may initiate stationary cycling with the orthosis on. Provide training in safe mounting/dismounting.
- Surgical patients should avoid excessive sweating in the orthosis until the wound is fully healed.

**6 Weeks Post-Injury:**

- Follow-up with physiotherapist.
- Repeat assessments: tendon palpation, Thompson test, and loading evaluation.
- Remove the next wedge (done by the patient or physiotherapist).
- Continue gait training. If discomfort arises, resume use of two crutches.
- The final wedge remains until orthosis discontinuation at 8 weeks post-injury.

**8 Weeks Post-Injury:**

- Follow-up and orthosis weaning.
- Repeat tendon assessment, Thompson’s test, and evaluate loading.
- Measure Achilles Tendon Resting Angle (ATRA).
- For surgical patients: continue scar evaluation and mobilization. Provide tape application guidance for scar maturation.
- Begin “Achilles Tendon Program II.” Seated exercises are performed barefoot.
- During standing/walking, indoors and outdoors, supportive shoes must be worn for four weeks post-orthosis.
- Distribute cork wedges for use in both shoes.
- Emphasize gait training with appropriate foot roll-off.
- Restoration of normal gastrocnemius activation is key to reducing pain, swelling, and optimizing function.
- Each step should promote natural tendon loading and circulation.
- Aim for symmetry in step length, stance phase, and muscle activation across joints.
- Continue using one or two crutches until gait is normalized.
- Identify and discuss risk factors (e.g., stairs, curbs, inclines, uneven terrain).
- Advise against walking as exercise; cycling is preferred to stimulate circulation. Compression stockings may also be beneficial.
- Driving should be avoided until coordination and strength are sufficient.

**8–12 Weeks Post-Injury:**

- Ongoing rehabilitation with gradual progression of load and speed, based on individual assessment.
- Focus on restoring automatic, normalized gait pattern.
- Crutches should be used until gait mechanics are optimal.
- Balance protection of healing structures with appropriately timed loading.
- Many patients benefit from more frequent physiotherapy visits following orthosis discontinuation.
- At 12-week orthopedic follow-up, include completed ATRS (Achilles Tendon Total Rupture Score) and activity level (Grimby scale).

**12 Weeks and Beyond:**

- Continue individualized rehabilitation. Progressively increase load with the goal of returning to pre-injury function.
- Stretching of the gastrocnemius-soleus complex should be avoided before 16 weeks post-injury, as elongation is a major risk for compromised recovery.
- For patients with ankle stiffness, introduce range-of-motion exercises in positions that minimize Achilles tendon stretch (e.g., knee flexed, foot supported).
- Outdoor cycling can typically begin after 12 weeks, starting cautiously.
- Total rehabilitation time ranges from 6–12 months.
- Sport-specific training may begin at 4–6 months if progress is satisfactory.
- Return to high-impact sport typically requires 9–12 months.
- Full tendon tensile strength is generally not regained until 12 months post-injury.

**Foot – Achilles Tendon Program I**
**Home Exercise Program**
**Sahlgrenska University Hospital – Occupational Therapy and Physiotherapy**

**Instructions:**
During the **first 6 weeks** while wearing the orthosis, **remove it 3–5 times daily** to perform these exercises.

**Important Considerations During Rehabilitation Following Achilles Tendon Rupture:**

There are two key risks that must be avoided:

- **Re-rupture** of the tendon
- **Tendon healing with excessive length**, which leads to decreased strength

To minimize these risks:

- **Avoid excessive stretching** of the tendon in the early phase.
- Stretching occurs when the foot is dorsiflexed (bends upward).

For the tendon to heal optimally and regain elasticity, **gentle and appropriate activation** is essential.

**Example setup**: Orthosis on the **left foot**, with **3 wedges** placed under the grey insole.

**Exercise 1**

**Ankle plantarflexion in prone or sitting**

**Position:**

- Lying face down with your feet hanging off the edge of a bed, **or**
- Sitting with the lower leg supported and knee straight

**Action:**

- Point the foot down (plantarflex), then slowly return to a relaxed position.

**Dosage:**
**3 sets of 20 repetitions**

**Exercise 2**

**Side-to-side ankle movement**

**Position:**

- Seated or lying face down as in Exercise 1

**Action:**

- Gently move the foot from side to side in a small arc.
- Keep the knees still.

**Dosage:**
**3 sets of 20 repetitions**

**Exercise 3**

**Supported seated heel raise in orthosis**

**Position:**

- Seated, with orthosis on
- Deflate the orthosis air cushions and open the front panel
- Hold the top of the orthosis with both hands for support

**Action:**

- Perform a **small heel lift**, keeping the **forefoot in contact with the sole**.
- Do **not grip with the toes**.
- This safely activates the calf muscle while the wedges protect the tendon from stretching.

**Dosage:**
**2 sets of 10 repetitions**

**General Guidelines:**

- All exercises should be **comfortable and pain-free** — **no stretching, tingling, or sharp pain**.
- **Always wear the orthosis when standing or walking**.
- It’s **normal** for the tendon and ankle area to be slightly **warmer and more swollen** than the uninjured side during healing.
- However, **progressive swelling, redness, or increasing warmth** may indicate **overuse** of the tendon and should be addressed.

**Foot – Achilles Tendon Program II**
**Home Exercise Program**
**Sahlgrenska University Hospital – Occupational Therapy and Physiotherapy**

### **Instructions:**

Perform the exercises **3–5 times per day**.

### **Circulation**

**1) Gentle ankle pumps**

- Pump the foot up and down softly.
- You may place a **roll under the knees** for support.
- The movement should be **pain-free** and **comfortable** for the tendon.
  **20 repetitions**

### **Activation**

**2) Foot cupping with relaxed toes**

- Gently form a dome shape with the foot while keeping the toes relaxed.
  **10 repetitions**

### **Mobility**

**3) Forward-backward weight shift**

- Keep the entire foot sole in contact with the floor.
- Move the knee and body weight forward and backward over the foot.
- On the final repetition, hold the end position for a few seconds.
  **10 repetitions**

### **Strength**

**4) Seated heel raise**

- Sit with the **entire forefoot on the floor**.
- You may rest your hands on your knees.
- Lift the heels to perform a **heel raise**.
  **20 repetitions**

**Seated foot jogging**

- Perform small alternating bouncing movements with the feet while seated.
  **20 repetitions**

**Heel raise**

- Repeat the seated heel raise again.
  **20 repetitions**

## **Standing Exercises (with shoes)**

**Instructions:** Perform standing exercises with **shoes on**.

**5) Lateral weight shifting (side-to-side)**

- Transfer weight from one leg to the other.
- Load the entire foot on the stance leg.
- Feel muscle activation, especially in the **gluteal region**.
  **10 repetitions**

**6) Gait preparation exercise**

- Stand with a **book or rolled towel under both heels** and lean against a wall.
- Alternate lifting and lowering each heel gently.
- Focus on **stable hips**, **relaxed toes**, and **fluid knees**.
  **20 repetitions**

**7) Gait training**

- Walk in a straight line, placing one foot directly in front of the other.
- Imagine walking on a **tightrope**.
- Walk **10 steps forward**, then **10 steps backward**.
- Use **crutches until you feel stable without them**.

### **Walking Guidelines:**

- **Aim to walk with a normal gait pattern**: heel strike, roll through, and even step length.
- Short steps are often better in the early phase.
- **Use crutches as long as needed**.
- Walk as much as necessary in daily life, but **do not walk for exercise** at this stage.
- Many post-injury issues are caused by **over-walking** during early recovery.

### **To Reduce or Prevent Swelling:**

- Elevate the foot while sitting.
- Perform **seated ankle pumps** and **toe spreading and gripping**.
- If needed, consider using a **compression sock**.
